# Pilot Study Demonstrates Tetramethylhexadecenyl Succinoyl Cysteine (TSC) Reduces Tretinoin‐Induced Erythema and Dryness in Human Subjects

**DOI:** 10.1111/jocd.70026

**Published:** 2025-02-07

**Authors:** Eduardo Pérez, José R. Fernández, Yasanuri Sato, Corey Fitzgerald, Karl Rouzard, Jason Healy, Masanori Tamura, Jeffry B. Stock, Tsuyoshi Ishii

**Affiliations:** ^1^ Signum Biosciences Monmouth Junction New Jersey USA; ^2^ Rohto Pharmaceutical Co. Ltd. Osaka Japan; ^3^ Department of Molecular Biology Princeton University Princeton New Jersey USA

To the Editor,

Isoprenylcysteine (IPC) molecules represent a novel, safe class of compounds with a broad range of anti‐inflammatory, antimicrobial, wound healing, and moisturizing properties. These molecules are primarily synthesized with two distinct long tail fatty acid chains attached to a cysteine residue, either possessing a 15‐carbon farnesyl or a 20‐carbon phytyl moiety. We previously reported that novel IPC compounds, diosodium tetramethylhexadecenyl succinoyl cysteine (TSC aka SIG1273) and acetylglutaminoyl farnesylcysteine (QFC aka SIG1191), possess several skin‐protecting properties, including the inhibition of UVA, UVB, Ni^+2^, toll‐like receptor, and T‐cell receptor‐induced inflammation [1, 2, 3]. Moreover, other farnesyl cysteine derivatives have been shown to reduce erythema when topically applied to human subjects [4], while phytyl cysteine–derived compounds have been shown to be well tolerated and reduce signs of aging and are effective in subjects with acne‐prone skin [5, 6]. Topical retinoids, such as all‐trans retinoic acid (tretinoin) and retinol, are commonly utilized in both the prescription and cosmetic space to treat acne and skin aging. Per Verified Market Reports, the global market size for tretinoin is currently valued at USD $7.64 billion and projected to surpass $10 billion by 2030. However, despite their effectiveness, the topical use of retinoids can cause burning, itching, stinging, and redness for consumers. Given their previous skin health–promoting activities, we sought to perform a pilot clinical study to assess whether TSC and/or QFC could effectively reduce tretinoin‐induced irritation and thus ameliorate the negative side effects that come with its use.

This study was a 5‐day efficacy evaluation with five subjects performed at a third party CRO (Validated Claim Support, Teaneck, NJ). There were four female and one male participant with a mean age of 35 years ranging from 31 to 42 years. No adverse events were observed. Four test sites on the subjects' backs were utilized: Test site 1—negative control (untreated), Test site 2—positive control (tretinoin 0.2%), Test site 3—(0.5% TSC + 0.2% tretinoin), and Test site 4—(0.5% QFC + 0.2% tretinoin). In addition to the actives, the only other ingredients used in each formulation were isopropyl palmitate (74.3%–74.8%) and pentylene glycol (25%). Test samples (0.1 mL) were applied topically under an occlusive patch at Days 0 and 1. No application was made on Days 3 or 4. Objective tolerance grading and clinical photography were captured from Day 1 to 4. Clinical results demonstrate that 0.5% TSC +0.2% tretinoin elicited a milder response and faster reduction in both dryness and erythema as compared to the positive control, tretinoin 0.2%, and 0.5% QFC + 0.2% tretinoin (Table 1; Figure 1). Increase in dryness persisted through the final evaluation day (Day 4) for the positive control, while 0.5% TSC + 0.2% tretinoin showed a decrease over the evaluation period (Table 1; Figure 1). A difference of 47% (mean cumulative scores) between 0.5% TSC + 0.2% tretinoin and tretinoin 0.2% was noted for dryness. Additionally, erythema in the 0.5% TSC + 0.2% tretinoin test site was reduced significantly by 68% (*p* = 0.010*) at Day 4 compared to tretinoin 0.2% (39%) and 0.5% QFC + 0.2% tretinoin (36%). The results from this pilot study suggest that the phytyl‐cysteine lipid tail (TSC) may be more effective in reducing tretinoin‐induced dryness and erythema than the farnesyl moiety, as QFC appeared to have little to no effect on this type of irritation, while TSC effectively improved dryness and reduced redness. Altogether, given these initial encouraging results, further study of TSC in a dose‐dependent manner in a larger, controlled study is warranted.

**TABLE 1:** Tolerance grading summary.

	Time point	0.2% Tret	0.2% Tret + 0.5% TSC	0.2% Tret + 0.5% QFC
		Mean ± SD	Mean ± SD	Mean ± SD
		% change^	% change^	% change^
		(*p*)	(*p*)	(*p*)
Assessment	Day 2	1.40 ± 1.34 N/A (N/A)	1.20 ± 1.30 N/A (N/A)	1.00 ± 1.22 N/A (N/A)
	Day 3	1.80 ± 0.84 29% (0.688)	1.10 ± 1.14 −8% (0.922)	1.40 ± 1.14 40% (0.670)
	Day 4	2.00 ± 0.71 43% (0.305)	1.00 ± 1.00 −17% (0.799)	2.20 ± 0.84 120% (0.109)
Mean of cumulative scores (% difference of 0.2% Tret + 0.5% TSC to 0.2% Tret)		1.50 (N/A)	0.93 (−47%)	1.35 (N/A)
	Day 2	2.30 ± 0.45 N/A (N/A)	2.50 ± 0.50 N/A (N/A)	2.50 ± 0.45 N/A (N/A)
	Day 3	2.10 ± 0.55 −9% (0.587)	1.70 ± 0.27 −32% (0.056)	2.30 ± 0.67 −8% (0.621)
	Day 4	1.40 ± 0.65 −39% (0.009*)	0.80 ± 0.45 −68% (0.010*)	1.60 ± 0.89 −36% (0.053)
Mean of cumulative scores (% difference of 0.2% Tret + 0.5% TSC to 0.2% Tret)		1.80 (N/A)	1.53 (−17%)	1.98 (N/A)

*Note:* Tolerance grading was performed by an expert grader using the following five‐point ordinal scales (half points will be included: 0.5, 1.5, 2.5, and 3.5) for dryness, erythema, and edema on Days 0–4. The TTEST uses the data in array1 (Baseline) and array2 (Subsequent TP) to compute a non‐negative *t*‐statistic using a paired two‐tailed distribution.

Abbreviations: SD, standard deviation; Tret, tretinoin; TSC, tetramethylhexadecenyl succinoyl cysteine; QFC, acetylglutaminoyl farnesylcysteine.

* Statistically significant (*p* ≤ 0.050), mean decrease = improvement.

**FIGURE 1:**
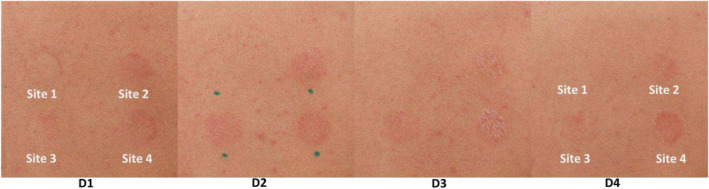
TSC, but not QFC, visually improves tretinoin‐induced irritation and dryness. Images of the scapular back were captured at Day 1 (D1) through Day 4 (D4) utilizing a Canon R5. Subjects were positioned on a stool resting their arms in front of them. The below images are for Subject #4. Site 1—baseline (untreated), Site 2—0.2% tretinoin (positive control), Site 3—0.5% TSC + 0.2% tretinoin, Site 4—0.5% QFC + 0.2% tretinoin.

## Author Contributions

E.P., J.S., Y.S., and J.F. designed the research study; T.I., K.R., C.F., Y.S., M.S., and E.P. analyzed the data; J.H. and M.T. contributed essential reagents or tools; and E.P. wrote the paper. All authors read and approved the final letter prior to submission.

## Ethics Statement

This study was conducted in accordance with the intent and purpose of Good Clinical Practice regulations described in Title 21 of the U.S. Code of Federal Regulations (CFR) and the Declaration of Helsinki and its later amendments.

## Conflicts of Interest

All authors of this letter are paid employees or consultants of Signum Biosciences or Rohto Pharmaceutical, which funded the study.

## Data Availability

The data presented in this study is available from the authors upon reasonable request.
